# Primary angiitis of the CNS (PACNS) and Behçet disease

**DOI:** 10.1186/s42466-019-0014-4

**Published:** 2019-03-26

**Authors:** Peter Berlit, Markus Krämer, for the Consensus Group, Marcel Arnold, Martin W. Baumgärtel, Peter Berlit, Thomas Gattringer, Bernhard Hellmich, Markus Krämer, Peter Lamprecht, Christof Specker, Andreas Steinbrecher

**Affiliations:** 1 Deutsche Gesellschaft für Neurologie, Reinhardtstr. 27 C, 10117 Berlin, Germany; 2 Neurologie, Alfried-Krupp-Krankenhaus Essen, Alfried-Krupp-Straße 21, 45131 Essen, Germany

**Keywords:** Primary angiitis of the CNS (PACNS), Reversible cerebral vasoconstriction syndrome (RCVS), Behçet disease, Neuro-Behçet, Isolated angiitis of the CNS

## Abstract

Cerebral vasculitis is a rare disorder but plays a major role in the differential diagnosis of stroke, encephalopathy and headache. This guideline was developed in order to support clinicians in the diagnosis and treatment of primary angiitis of the CNS (PACNS) and Neuro-Behçet. It is based on a medline research and was developed in a modified Delphi process and approved by the involved societies.

This article is an abridged and translated version of the guideline published in *DGNeurologie*: Berlit, P. & Krämer, M. DGNeurologie (2018) 1: 17. 10.1007/s42451-018-0001-y

## Most important recommendations at a glimpse

- The clinical diagnosis of primary angiitis of the CNS (PACNS) using MRI and DSA, but without biopsy leads to frequent misdiagnoses.
- An unremarkable MRI and normal CSF findings make PACNS unlikely.
- The most important differential diagnosis of PACNS is the reversible cerebral vasoconstriction syndrome (RCVS) with angiographic changes “typical of vasculitis”. The recurrent thunderclap headaches of RCVS are helpful in the differential-diagnostic delineation.
- The induction therapy of PACNS consists of the combined administration of glucocorticoids (GC - 1 mg/kg body weight (BW) prednisolone) and cyclophosphamide (CYC), usually given i.v. in doses of 0.75 g/m2 body surface (BS) monthly for 6 months, followed by maintenance therapy with azathioprine (AZA), mycophenolate mofetil (MMF) or methotrexate (MTX).
- Alternatively, PACNS may be treated with GC and rituximab.
- Behçet Disease is a vasculitis of unknown etiopathogenesis which affects arteries and veins of different sizes. It is associated with circulating immunocomplexes and the haplotype HLA-B51
- Neurological involvement occurs in 10–40% of all Behçet patients, an average 5 years after onset of mucosal, skin and eye manifestations in the 3rd and 4th decade of life. Differentiation is made depending on the distribution pattern in parenchymatous (80%) and non-parenchymatous (vascular, 20%) Neuro-Behçet.
- Therapy follows consensus recommendations, with GC (500–1000 mg methylprednisolone) over 5–7 days and oral tapering over 2–3 months.
- Long-term interval therapy is conducted with low-dose GC combined with a steroid-sparing immunosuppressive (AZA, chlorambucil or MTX).
- In the treatment of neurological complications, infliximab, tocilizumab or alemtuzumab may be tried. Sinus thromboses are anticoagulated.

## Primary angiitis of the CNS (PACNS)

This article is an abridged and translated version of the guideline published in *DGNeurologie*: Berlit, P. & Krämer, M. DGNeurologie (2018) 1: 17. 10.1007/s42451-018-0001-y

## Definition and classification

Primary angiitis of the CNS (synonyms: isolated angiitis of the CNS, primary CNS-Vasculitis) (PACNS) is a rare disease affecting all age groups, which is probably too-frequently diagnosed without biopsy confirmation. The symptoms are unspecific and cannot be discriminated from other diseases [3]. An encephalopathy with cognitive and affective features, persistent headache as well as multifocal symptoms with recurrent ischemias or bleeding and epileptic seizures may occur. The spinal cord may also be affected [31], but PACNS with exclusively spinal manifestion is rare [11].

This is an etiologically unclear inflammation of the small and middle vessels exclusively in the central nervous system, whereby the inflammation shows histological evidence of a granulomatous vascular wall inflammation with and without ß-amyloid deposit, with transmural lymphocytic infiltrates or fibrinoid necroses of the vascular wall [23].

Two variants are differentiated: a Small-Vessel Variant (SV-PACNS) with a high risk of recurrence and a Medium-Vessel Variant (MV-PACNS) with a better prognosis [22]. It is to be expected that SV-PACNS will often present with elevated acute phase serology, normal angiography, but positive findings in the cerebral biopsy. Especially when elevated cerebrospinal fluid (CSF)-protein and lesions with gadolinium uptake are present, the SV-PACNS appears to have a favorable prognosis [33].

## Diagnostics

Since, as a rule, this is a disease with subacute to chronic course and a false-positive diagnosis of PACNS may have fatal consequences, differential diagnostics should always rule out involvement of systemic vasculitis, vasculitis with a different underlying disease (such as infection) or a different type of disease (such as the reversible cerebral vasoconstriction syndrome - RCVS, the Susac Syndrome or Moyamoya Angiopathy) [3, 6]. Exclusion of differential diagnoses is particulary important due to the difficulty in positive diagnosis of a PACNS with the dilemma of the possibility that cerebral biopsy may even be negative [4] (Table 1).

**Table 1:** Diagnostic criteria for the Diagnosis of a PACNS (modified after [9])

Definitive Diagnosis of a PACNS	The definitive diagnosis of a PACNS can only be made with histologic confirmation.
Probable Diagnosis of a PACNS	When no bioptic confirmation is possible, the probable diagnosis of a PACNS can only be made whith typical angiographic and MRI findings, in addition to CSF findings consistent with PACNS.

### Anamnesis and clinical examination

Anamnesis should include a detailed family history and the question for eliciting factors (drugs, among others). In the clinical examination, attention should be paid to rheumatological and skin symptoms such as a livedo racemosa or angiokeratoma, evidence of dysmorphias or a disease of the connective tissues, such as hyperlaxidity of the skin and joints. The presence of all of these queried and examined symptoms makes PACNS unlikely. Hereditary vasopathy, Fabry disease, disorders of connective tissues and infectious origin must be given special attention in the differential diagnose.

### Magnetic resonance imaging (MRI)

PACNS is very unlikely if MRI is unremarkable. Multifocal lesions in the white matter are found MR-tomographically, the MR angiography does not always reveal remarkable findings (47–59%) [32]. PACNS may present as a mass lesion, and is then, naturally, very often misinterpreted as a tumor and diagnosed bioptically [25]. MR imaging should include diffusion-weighted images and ADC maps to demonstrate ischemic changes varying in age, gradient echo sequences to reveal microbleeds, and gadolinium administration, since lesions with gadolinium uptake and leptomeningeal enhancement have been described [9, 30]. High-resolution, contrast-enhanced, flow-compensated and lipid-saturated MR images of the vascular wall (black blood imaging) are of only limited value in the detection of vasculitis, since this or similar contrast behavior can also be seen in aneurysms, arteriosclerosis and vascular spasms [26].

### Serum and CSF findings

Erythrocyte sedimentation rate [ESR] and C-reactive protein [CRP] may be elevated in serum, but in less than one-quarter of the patients [31]. CSF findings include lymphomonocytic pleocytosis or elevated protein in 90% [3, 6, 32], normal CSF findings make PACNS unlikely. The elevations of cell count and protein are only slight to moderate. With pleocytosis > 250/μl other, especially infectious diseases should be considered [9]. CNS-vasculitides themselves can be caused by a number of infections and must then undergo appropriate antibiotic or antiviral treatment. Varicella-Zoster-Virus (VZV)-vasopathy, which occurs frequently especially in children, should be ruled out by PCR and antibody evidence in CSF and blood [27].

### Cerebral angiography

Conventional cerebral digital subtraction angiography (DSA) was long considered the diagnostic gold standard, but it can only offer support in the diagnosis of PACNS. The sensitivity of DSA is, however considerably higher than that of MR angiography, but in light of the lower risk of MRI, this has taken a firm place in diagnostics. In PACNS, different correlations between MR angiography and DSA have been described (56% versus 78%) [21]. Angiography can be negative if the small vessels (e.g. < 500 μm) are affected [30]. The sensitivity of DSA is reported as between 50 and 90%. It must be remembered especially that in RCVS the angiographic changes often appear “typical of vasculitis”. In RCVS, mild pleocytosis in CSF is possible [18], but the characteristic recurrent thunderclap headaches of RCVS are helpful in the differential-diagnostic delineation [34]. Microaneurysms are a typical finding of polyarteritis nodosa [9], bilateral collateral nets with stenoses or occlusions of intracranial ICA, MCA and ACA are the hallmark of moyamoya angiopathy [7, 19]. Divry-van-Bogart Syndrome can only be excluded by DSA [8].

### Biopsy

All patients with a well-founded suspicion of PACNS should undergo leptomeningeal and cerebral biopsy after thorough differential diagnostic examinations; stereotactic biopsies are recommended only for mass lesions. The clinical diagnosis of PACNS using MRI and DSA, but without biopsy leads to frequent misdiagnoses [3, 6]. The risk of a falsely-indicated long-term immunosuppression is generally considered to be higher than the risk of cerebral biopsy [5]. The bioptic exclusion of differential diagnoses, especially primary CNS lymphomas, is important. Harvesting of a leptomeningeal and parenchymatous biopsy should be made whenever possible in an area affected by MRI or angiography. The preferred biopsy site is the non-dominant hemisphere, outside eloquent areas.

False-negative biopsies are not infrequent and are a particular diagnostic dilemma. It must be remembered that histology confirms only the vasculitis as such, but not its etiology. An infection, for example, must be ruled out by additional microbiological diagnostics in tissue or as part of CSF diagnostics. There are three different histological patterns in PACNS: granulomatous with multinuclear cells, often also with Beta-Amyloid deposits (58%), lymphocytic vasculitis (28%) or necrotizing vasculitis with fibrinoid necroses and frequently intracerebral bleeding (14%) [33]. In the inflammatory variants of cerebral amyloid angiopathy (CAA), differentiation is made between cases with perivascular cellular infiltrates (CAA-related inflammation – CAA-ri) and those with transmural inflammation of the vessel wall (A-beta-related Angiitis - ABRA).

### Exclusion of other diseases

There is a large heterogeneous group of differential diagnoses which can imitate the clinical picture or individual findings. The exclusion of differential diagnoses is of uttermost importance for the diagnosis of PACNS. A German study found a total of 15 differential diagnoses in 44 of 69 patients referred with the diagnosis PACNS [3]. Among these were diseases which could be treated with similar therapeutics, like systemic vasculitides, but there were also diseases requiring other treatments, such as multiple sclerosis, moyamoya disease and RCVS.

Numerous pathogens can lead to an inflammatory vasopathy or pathogen-related vasculitis (VZV, HIV, Hepatitis C, tuberculosis, borreliosis and syphilis [20]). In particular, a VZV-vasopathy should be ruled out. Both during the initial manifestation (varicella) and during the reactivation (zoster), stroke may occur on the basis of inflammatory vascular changes. While infestation of the large vessels usually affects the intracranial segment of the ICA or the MCA and is often associated with varicella or an ophthalmic zoster, a VZV vasopathy of the small vessels can occur without cutaneous manifestation. In this case, the differential diagnosis to PACNS is more difficult, making VZV-PCR in CSF and determination of the VZV-antibody index (serum/CSF) obligatory [27, 29].

Important differential diagnoses of PACNS are presented in Table 2.

**Table 2:** Differential diagnoses of PACNS [6, 9]

Other inflammatory diseases	
− Autoimmune encephalitides	
− Susac-Syndrome	
Non-inflammatory diseases	
− Atherosclerosis	
− Neurofibromatosis	
− Fibromuscular Dysplasia, Ehlers-Danlos IV, Marfan-Syndrome	
− Genetic microangiopathies (COL4A1, CTC1, TREX)	
− CADASIL, CARASIL	
− Leukodystrophies	
− MELAS	
− Moyamoya angiopathy	
− Fabry disease	
− Sneddon syndrome	
− Hypercoagubility	
Demyelinating diseases	
− Multiple Sclerosis	
− ADEM	
− NMOSD	
CNS-Involvement in Systemic Vasculitis	
− Large vessel vasculitides: Giant cell arteriitis, Takayasu Arteriitis	
− Medium size vessel vasculitides: Polyarteriitis nodosa, Kawasaki disease	
− Small vessel vasculitides: ANCA-associated vasculitides (Granulomatosis with polyangiitis, microscopic polyangiitis, eosinophilic granulomatosis with polyangiitis) Immunocomplex-associated diseases (IgA-Vasculitis – Schönlein-Henoch Purpura, cyoglobulinemic vasculitis)	
Involvement in Infections	
− Embolisms of subacute bacterial endocarditis	
− VZV-Vasopathy	
− HSV, HIV, Hepatitis B, C, Parvoviruses Borreliae Lues, Tuberculosis, Rickettsias, fungi, protozoans	
− parainfectious syndromes	
Involvement in Systemic Diseases	
− Systemic Lupus erythematodes	
− Sjögren-Syndrome	
− Sclerodermia	
− Neurosarcoidosis	
− Neurobehçet	
Reversible Cerebral Vasoconstriction Medication Syndromes or Drug-induced Malignant Syndromes Diseases	
− Primary CNS-Lymphoma	
− Intravascular Lymphoma	
− Lymphomatoid Granulomatosis	

## Therapy

As a rule, when suspected PACNS is diagnosed, a “blind” treatment with GC or immunosuppressives should be avoided. The published (empirical) criteria require at least the histopathological exclusion of infectious diseases prior to initiating immunosuppressive therapy [6]. The clinical presentation and also the neuroradiological and CSF findings may be imitated by infectious diseases, such as endocarditis with recurrent septic embolisms or pathogen-related vasculitides [5]. In such cases, “blind treatment” may lead to the patient’s death [3]. Prior to initiating treatment, therefore, cerebral biopsy and the infectiological examination of the CSF are the clinical standard [6].

The decision in the individual case must be made depending on the dynamics of the disease. In an unclear diagnostic constellation (such as negative biopsy with otherwise typical findings) and a clinically-stable situation, follow up including MRI and CSF should be considered, for example after 3 months. Depending on the dynamics of the vascular changes (e.g. reversible in RCVS, fairly stable in atherosclerosis, more likely increasing in PACNS), a decision concerning immunosuppression can then be made [3]. In the cohort of the Mayo-Clinic, high dose steroids, followed by oral GC administration were applied [32].

The therapy of choice consists of the combined administration of GC (1 mg/kg BW prednisolone) and cyclophosphamide (CYC), usually given i.v. in doses of 0.75 g/m2 BS monthly for 6 months. Among the 163 patients at the Mayo Clinic (Rochester, USA), steroid therapy alone produced a responder rate of 86%, but an odds ratio (OR) for recurrence of 2.9 [32]. The combined administration of GC and CYC resulted in a similar success rate, but a significantly lower recurrence rate. The prognosis was less favorable if large vessels were affected (OR 6.1) and if stroke was already present at the start of therapy (OR 3.3).

Due to the side effects of CYC, many authors recommend a 6-month CYC therapy followed by maintenance therapy with AZA, MMF or MTX [9].

In analogy to the studies in ANCA-associated vasculitides, in which it could be shown that RTX is just as effective as CYC, RTX has been increasingly used in PACNS in light of the good side effects. The required length of treatment is unclear. In individual cases, Infliximab, Tocilizumab or Etanercept was also used. Treatment monitoring is done clinically and using MRI, and where appropriate via CSF and DSA. In the inflammatory variants of CAA, especially ABRA, the prognosis appears to be even better with immunosuppressive therapy than in PACNS.

## Behçet disease

Behçet Disease is a vasculitis of unknown etiopathogenesis which affects arteries and veins of different sizes. In the CHCC nomenclature, it is therefore termed vasculitis of variable vessel size [15]. It is associated with circulating immunocomplexes and the haplotype HLA-B51 [14]. Infectious triggers, autoimmune-mediated processes, prothrombotic anomalies of the coagulation system and a genetic predisposition are discussed. The annual incidence is 1 in 500,000 inhabitants in Germany, but 300–500 of 100,000 in Turkey. In the Netherlands, a prevalence of 1/100,000 was found among Caucasian inhabitants, 71/100,000 in inhabitants of Turkish origin and 39/100,000 in ethnic Moroccan inhabitants. Men are twice as often and more severely affected than women. The main manifestation age is between 20 and 40 years [12].

### Clinic and diagnostics

Recurrent oral and genital ulcerations, eye inflammations (uveitis) and skin changes are the guiding symptoms. Only 3% of the patients do not show oral ulcerations. Genital ulcerations are found in 60–90% of the patients, manifest in the scrotum or labiae and - in contrast to oral ulcers - leave scars. In the eyes, there are anterior or posterior uveitis, vitreous body infiltrates or retinal vasculitis. Among the skin changes are erythema nodosum, pseudo-folliculitides or papulopustular lesions. A non-specific pustulous reaction 24–48 h after local needle prick is termed a positive pathergy test. The developing pustula is sterile.

Basically, Behçet Disease is a multisystem disease of vasculitic origin, in which, in addition to the skin/mucosae and eyes, the joints (mono- or oligoarthritis), the gastrointestinal tract (ulcerations of the mucosa in the ileum or coecum), the lungs (pulmonary arterial arteritis) and the aorta or vessels of the extremities (thrombophlebitis, arteritis with development of pseudoaneurysms) may be affected (Table 3).

**Table 3:** Diagnostic Criteria (International Team for the Revision of the International Criteria for Behcet’s 2014)

Criteria	Points
Eye involvement (Uveitis/Iritis with hypopyon, Retinitis)	2
Genital ulcerations (usually heals with scars)	2
Oral ulcerations (usually 3 x per year, no sequelae)	2
Skin lesions (Erythema nodosum, folliculitis, sterile pustulae)	1
Neuro-Behçet (no isolated headache)	1
Vascular manifestation (venous or arterial thromboses, aneurysms)	1
Positive pathergy test (optional)	1

4 Points: possible, 5 Points: very probable, 6 Points: almost certain diagnosis (94% sensitivity, 90% specificity)

### Neuro-Behçet

Neurological involvement occurs in 10–40% of all Behçet patients [16], an average 5 years after onset of mucosal, skin and eye manifestations in the 3rd and 4th decade of life. Differentiation is made depending on the distribution pattern in parenchymatous (80%) and non-parenchymatous (early vascular, 20%) Neuro-Behçet.

Although vasculitis is the pathological hallmark in various lesions (skin, genitals, eye) and vasculitis with involvement of large vessels is confirmed, vasculitis is not regularly found in the CNS. A mild-intensity chronic lymphocytic or neutrophilic meningoencephalitis and multifocal necroses in the brain stem and basal ganglia have been described [17]. Motoric deficits with spastic signs and brain stem symptoms, as well as distinctive mental features in the form of disruptions in memory and attention span are guiding symptoms of parenchymatous Neuro-Behçet [16]. The onset of symptoms is usually acute, the course runs in episodes. MRI shows extensive lesions with contrast uptake, preferred sites in the basal ganglia or in the brain stem reaching toward the diencephalon. These lesions are not limited to vascular territories and may cause brain stem atrophy. In 10–20%, the spinal cord is also affected. Aseptic meningitis and patients with purely psychopathological distinctive features are less frequent. In the CSF, at least half the patients present with pleocytosis and increased protein. Usually there is lymphocytic, less often a mixed-cell or primarily granulocytic pleocytosis (0–485, median 30/μl) or isolated protein elevations. While in 70% of the cases there is a pathological IgG-Index, oligoclonal banding is often only temporarily present. In the laboratory analysis, the HLA-B-51 status may be helpful: In Morbus Behçet, 40–80% of the patients present this HLA characteristic, while it can be found in up to 24% of healthy Turkish patients and in up to 8% of healthy German patients [12]. It may be difficult to distinguish multiple sclerosis from Neuro-Behçet. The main symptoms of non-parenchymatous Neuro-Behçet (20% of the total group) are intracranial hypertension, sinus- or venous thromboses and aseptic meningitis [16]. Sinus- or venous thromboses are visible in MR-angiography, tissue lesions in the MRI.

### Therapy

Therapy follows consensus recommendations, since there are no large therapy studies [16]. Administration of GC (500–1000 mg methylprednisolone) over 5–7 days is considered the therapy of choice in an acute disease episode; oral tapering over 2–3 months is expected to prevent early recurrences. Many patients with Neuro-Behçet require long-term interval therapy with low-dose GC. Typically, this treatment is combined with a steroid-sparing immunosuppressive, whereby AZA, chlorambucil and MTX alone or in combination have been tried in smaller studies [28].

At any rate, 38 of the examined 40 patients with Neuro-Behçet responded to CYC in a study by Aid Ben Haddou. CYC was administered with GC at 600 mg/m2 BS on days 1, 2, 4, 6, and 8. Then a bolus of 600 mg/m2 BS every 2 months for 2 years followed [2].

Therapy with *Ciclosporin A,* which has high effectivity in the treatment of ocular lesions, is not recommended in the treatment of neurological complications, since the CNS side effects which are occasionally observed under Ciclosporin A are very difficult to differentiate from the symptoms of the underlying disease. Sinus thromboses are anticoagulated, whereas there is no consensus concerning peripheral leg vein thromboses. Here, immunosuppression appears to be more important than anticoagulation [1]. For *Infliximab,* efficacy and safety in neurological involvement was shown in a Phase 3 study. Infliximab was administered at 5 mg/kg BW 0, 2, 6 and then every 8 weeks. If the response was incomplete at week 30, the dose was increased to 10 mg/kg BW [13]. *Tocilizumab* was beneficial in uveitides which were unresponsive to interferone and anti-TNF-Alpha therapy [10]. *Alemtuzumab* is an option in severe, therapy-refractive Morbus Behçet [24].
